# The Texas Health Train

**Published:** 1914-09

**Authors:** Ralph Steiner


### The Texas Health Train.

The following letter from Dr. Steiner came in answer to a query from the Texas Medical Journal as to the purpose, plans and extent of the work to be accomplished by the health train. This is a most excellent work, and we bespeak for Dr. Steiner the active co-operation of every doctor in Texas:

*Mrs. F. E. Daniel, Austin, Texas.*

DEAR MRS. DANIEL: It is the intention of the Health Department to start the health car on its journey of instruction about September 10th. The purpose of this exhibit is to disseminate information concerning the cause, nature, extent and prevention of communicable disease and to arrange for free lectures and health exhibits, and to print and distribute, free of cost to the people, bulletins, pamphlets, circulars, leaflets, cards and other printed matter, printed in English, Spanish and German, containing useful information for the protection of the individual and the public health. We will send this exhibit over the different railroad lines in the State, and will cause it to be displayed in the different cities and towns along the line. With the display of the exhibit there will be given free lectures and talks to the people, illustrated, where possible, with stereopticon and moving pictures, and printed matter containing useful information pertaining to the protection of health and prevention of disease will be distributed.

The Health Department will encourage the organization of county and city societies and committees for the prevention of disease and protection of the public health, and will co-operate with any corporation organized under the laws of Texas for benevolent and charitable purposes in the movement to disseminate information concerning the cause, nature, extent and prevention of communicable disease.

Dr. O. H. Judkins has been appointed director of the exhibit.

Yours truly,

RALPH STEINER,
State Health Officer.

---

HIS FATAL SLIP.—Joe Coyne tells a story about a seedy-looking individual who got into conversation in a railway carriage.

"Ah, sir," he said sadly. "I've seen changes. I was once a *doctor* with a large practice, but owing to one little slip my patients began to leave me, and now I'm just living from hand to mouth."

"What was the slip?" I asked.

"Well, sir," he replied, "in filling a death certificate for a patient who had died I absent-mindedly signed my name in the space headed, 'Cause of Death.'"

